# Skin Cancer Prevention and Antiaging: Role of Nicotinamide

**DOI:** 10.3390/ijms27114918

**Published:** 2026-05-29

**Authors:** Francesco Moro, Annarita Silvia Irene Panebianco, Valeria Bartolocci, Alessio Capone, Antonio Di Guardo, Mariafrancesca Hyeraci, Giuseppe Paolo Antonio Gemma, Giovanni Di Lella, Laura Colonna, Francesco Ricci, Elena Dellambra, Luca Fania

**Affiliations:** 1 Dermatology Unit, IDI-IRCCS, 00167 Rome, Italy; f.moro@idi.it (F.M.); a.panebianco@idi.it (A.S.I.P.); g.dilella@idi.it (G.D.L.); l.colonna@idi.it (L.C.); f.ricci@idi.it (F.R.); 2 Dermatology Clinic, Department of Medical and Cardiovascular Sciences, Sapienza University of Rome, 00161 Rome, Italy; giuseppe.gemma@uniroma1.it; 3 Molecular and Cell Biology Laboratory, IDI-IRCCS, 00167 Rome, Italy; v.bartolocci@idi.it (V.B.); a.capone@idi.it (A.C.); e.dellambra@idi.it (E.D.); 4 Department of Molecular Medicine, University of Padua, 35122 Padua, Italy; mariafrancesca.hyeraci@unipd.it; 5 Department of Life Science, Health, and Health Professions, Link University of Rome, 00165 Rome, Italy

**Keywords:** nicotinamide, NAD^+^ metabolism, photoaging, actinic keratosis, keratinocyte carcinoma

## Abstract

Nicotinamide (NAM), the amide form of vitamin B3, has gained increasing attention in dermatology due to its potential role in both skin aging and non-melanoma skin cancer (NMSC) prevention. This review summarizes the biological rationale and current clinical evidence supporting the use of NAM and other NAD^+^ precursors in photoaging and cutaneous carcinogenesis. Chronic ultraviolet exposure induces DNA damage, oxidative stress, inflammation, immune dysregulation, and extracellular matrix remodeling, linking photoaged skin to increased susceptibility to actinic keratoses (AKs), squamous cell carcinoma (SCCs), and basal cell carcinoma (BCCs). Through the NAD^+^ salvage pathway, NAM contributes to the maintenance of intracellular NAD^+^ pools, thereby influencing energy metabolism, DNA repair, mitochondrial function, redox homeostasis, and the activity of NAD^+^-dependent enzymes. Preclinical studies indicate that NAM enhances DNA repair, reduces oxidative stress and inflammatory signaling, supports autophagy and mitophagy, and improves epidermal barrier function and extracellular matrix integrity. Clinically, the strongest evidence for anti-aging effects concerns topical NAM, which consistently improves wrinkles, texture irregularities, pigmentation, and barrier function. Oral NAM has demonstrated chemopreventive activity in high-risk patients with previous NMSC, particularly by reducing the incidence of new SCCs and AKs during active treatment. However, despite a strong mechanistic rationale, current evidence remains heterogeneous, and additional long-term, skin-focused clinical trials are needed to better define efficacy, safety, optimal dosing strategies, and patient selection.

## 1. Introduction

Non-melanoma skin cancers (NMSCs), including basal cell carcinoma (BCC), squamous cell carcinoma (SCC), and actinic keratoses (AKs), are the most common age-related cutaneous malignancies [1,2]. AKs are lesions that develop on chronically sun-exposed skin and may progress to invasive SCC. Although these tumors are generally curable when detected early, their clinical impact remains substantial due to high incidence, frequent recurrence, and, in the case of SCC, the potential for local invasion and metastasis [1,2]. Ultraviolet (UV) radiation is the primary environmental factor linking cumulative tissue damage to both skin aging and carcinogenesis [1,2]. The skin provides a unique model in which aging and carcinogenesis can be studied simultaneously. Both processes are often driven by chronic environmental exposures, and many molecular pathways underlying senescence and tumor development overlap. UV radiation not only induces DNA mutations but also triggers extracellular matrix remodeling, oxidative stress, immune dysregulation, and chronic inflammation. Consequently, photoaged skin reflects the cumulative molecular and structural damage from prolonged UV exposure, rather than merely cosmetic changes. Chronically photodamaged skin carries an increased risk of developing skin cancer [3].

Current evidence supports the concept of chronic photodamage as a progressive biological continuum linking photoaging and skin carcinogenesis. Repeated UV exposure induces cumulative molecular alterations extending beyond direct mutagenesis, including mitochondrial dysfunction, extracellular matrix degradation, epigenetic dysregulation, chronic low-grade inflammation (“inflammaging”), cellular senescence, and local immunosuppression. Together, these processes contribute to field cancerization and create a permissive microenvironment for tumor initiation and progression [4,5]. Importantly, several of these mechanisms are shared across different forms of skin cancer, including melanoma, although the strongest clinical evidence supporting nicotinamide-based prevention currently concerns NMSCs.

Prevention of photoaging and skin tumors primarily relies on behavioral interventions and topical photoprotection. However, adherence to regular sunscreen use is often inconsistent, particularly in individuals with high cumulative UV exposure. This has led to growing interest in systemic photoprotective strategies that counteract the biological effects of UV radiation, including DNA damage, oxidative stress, inflammation, and local immunosuppression. Among these agents, nicotinamide (NAM) has emerged as a molecule of considerable interest. Beyond its role as a vitamin supplement, NAM has demonstrated potential in aging research, metabolic disorders, neurodegeneration, and skin cancer biology, and is increasingly studied for chemoprevention in tissues exposed to environmental stressors [3,6]. However, systemic interventions should be regarded as complementary to, rather than a replacement for, topical photoprotection [7]. UV-induced oxidative stress, DNA damage, and mitochondrial dysfunction also contribute to melanomagenesis. Thus, NAD^+^ depletion may represent a shared metabolic vulnerability across both NMSC and melanoma. However, the strongest clinical evidence supporting NAM currently concerns NMSC [8], whereas evidence for NAM-based prevention in melanoma remains insufficient [9,10].

Given the overlapping pathways of photoaging and carcinogenesis, this review provides a detailed analysis of the molecular mechanisms influenced by NAM and its dual potential in mitigating skin aging as well as preventing tumor development.

## 2. NAM and Nicotinamide Adenine Dinucleotide (NAD^+^) Metabolism

Nicotinamide (NAM), or Niacinamide, is an amid member of the vitamin B3 family, which includes nicotinic acid (NA), nicotinamide mononucleotide (NMN), and nicotinamide riboside (NR). NAM is a primary precursor of nicotinamide adenine dinucleotide (NAD^+^), a central cofactor in cellular redox reactions essential for ATP production and catabolic processes, as well as a co-substrate for NAD^+^-consuming enzymes [11,12].

Functionally, NAM indirectly supports energy metabolism and redox homeostasis by sustaining intracellular NAD^+^ pools and fueling NAD^+^-dependent reactions involved in glycolysis, the tricarboxylic acid cycle (TCA), oxidative phosphorylation (OXPHOS), and antioxidant defense mechanisms. Adequate NAD^+^ levels derived from NAM are required to maintain the balance between oxidized and reduced NAD^+^ cofactors (NAD^+^/NADH) and indirectly support the NADP^+^/NADPH redox couple, thereby regulating ATP production and cellular responses to oxidative stress. Beyond its metabolic role in energy production, NAM also modulates key signaling pathways by acting as a feedback regulator of NAD^+^-consuming enzymes involved in DNA repair, gene expression modulation, and metabolic regulation [11,12].

### 2.1. NAD^+^ and Energy Production

**Figure 1:**
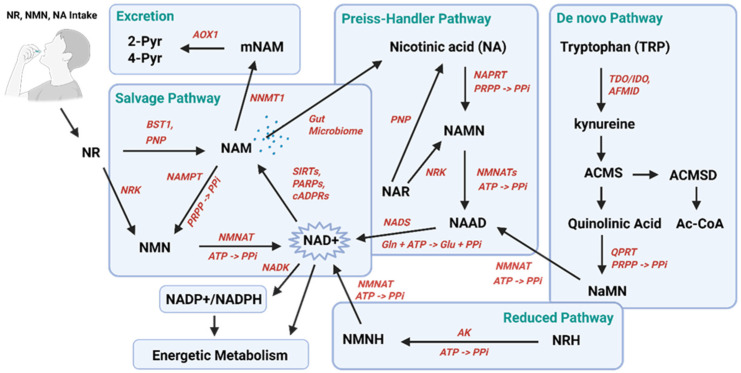
Nicotinamide and NAD participate in Energetic Metabolism and Enzymatic regulation. NAD+ is degraded by NAD+-consuming enzymes or enters the catabolic pathways for energy production, acting as an electron-transfer cofactor in redox reactions. The Salvage pathway is the predominant route for NAD regeneration from NAM, a key step controlled by NAMPT, while Preiss–Handler and De novo pathways converge from dietary uptake into NAAD, consequently transformed into NAD+ by NADS. Vitamin B3 analogs uptake promotes beneficial effects in vivo and in vitro, boosting energetic metabolism through the Salvage Pathway.

NAD^+^ can be synthesized through multiple interconnected pathways (Figure 1). The “de novo (kynurenine) pathway” generates NAD^+^ from dietary precursors such as tryptophan and converges with the “Preiss–Handler pathway”, which starts from nicotinic acid (NA). In both routes, deamidated intermediates are processed to form nicotinic acid adenine dinucleotide (NAAD), which is subsequently converted into NAD^+^ by NAD synthase (NADS). Conversely, the “salvage pathway” recycles amidated precursors, enabling the reutilization of NAM released by NAD^+^-consuming enzymes [13,14]. At the cellular level, NAM plays a pivotal role in the NAD^+^ salvage pathway, which represents the predominant route for NAD^+^ synthesis in mammals [15]. Upon NAD^+^ consumption, recycled NAM can re-enter the pathway, making NAM metabolism tightly integrated with NAD^+^ homeostasis and cellular energy balance. In the salvage route, NAM undergoes ATP-dependent conversion to nicotinamide mononucleotide (NMN) by NAM-phosphoribosyltransferase (NAMPT), the rate-limiting enzyme that uses 5′-phosphoribosyl-1-pyrophosphate (PRPP) as substrate. NMN is then adenylated to form NAD^+^ via NMN-adenylyltransferases (NMNATs), an ATP-consuming enzyme class that is also involved in the generation of NAAD from nicotinic acid mononucleotide (NAMN) in the Preiss–Handler pathway. In addition, NAM can be derived from nicotinamide riboside (NR). NR may be hydrolyzed to NAM by bone marrow stromal cell antigen 1 (BST1) and can also be further metabolized by the gut microbiota to NA [16]. NR can also be processed by purine nucleoside phosphorylase (PNP). However, in mammals, the predominant pathway for NR metabolism involves its phosphorylation to NMN by nicotinamide riboside kinases (NRKs) [17,18].

Beyond biosynthesis, NAM arises as a byproduct of numerous NAD^+^-consuming reactions (see Section 2.2), functionally linking NAD^+^ turnover to its own recycling within the metabolic network [12]. Reduced nicotinamide mononucleotide (NMNH) and reduced nicotinamide riboside (NRH) have also been recently described as precursors participating in NAD^+^ synthesis through a salvage “reduced pathway” [19]. NAD^+^ can also be phosphorylated to NADP^+^ by NAD kinase (NADK), expanding its redox and biosynthetic functions [20]. By sustaining intracellular NAD^+^ pools, these pathways directly support redox reactions driving glycolysis, the TCA, and OXPHOS, thereby contributing to ATP production.

NAM can undergo degradation, which contributes to the regulation of its intracellular and systemic availability. In hepatocytes, cytochrome P450 enzymes convert NAM into nicotinamide-N-oxide (N-Ox), whereas nicotinamide N-methyltransferase (NNMT) catalyzes its methylation to form 1-methyl-NAM (mNAM). mNAM is subsequently oxidized into 1-methyl-2-pyridone-5-carboxamide (2-Pyr) and 1-methyl-4-pyridone-5-carboxamide (4-Pyr) by detoxifying enzymes such as aldehyde oxidase (AOX1). mNAM and 2-Pyr represent the major urinary metabolites [11]. Through this degradative pathway, NAM may be withdrawn from the salvage pathway, thereby influencing NAD^+^ replenishment and overall metabolic balance.

### 2.2. NAD^+^-Consuming Enzymes

NAD^+^ serves as a co-substrate for several NAD^+^-consuming enzyme families, including sirtuins (SIRTs), poly-ADP-ribose polymerases (PARPs), and Cyclic ADP-ribose Synthases (cADPRSs) [21] (Figure 1). These enzymes cleave NAD^+^ during catalysis, generating NAM and triggering signaling processes that regulate genomic stability, energy metabolism, and cellular physiology. They are active in multiple tissues, including the skin, where NAD^+^ availability influences their activity [22].

SIRTs (SIRT1–7) are NAD^+^-dependent deacylases that regulate chromatin structure and protein function by removing acetyl or succinyl groups from histones and non-histone proteins. These reactions use NAD^+^ as a co-substrate, producing NAM as a byproduct, thereby linking post-translational modifications to cellular NAD^+^ metabolism [23,24,25]. NAM acts as a non-competitive inhibitor of SIRTs via transglycosylation, forming a ternary complex that prevents subsequent deacetylation events [26]. Accumulation of NAM creates a negative feedback loop that limits enzymatic activity if recycling to NAD^+^ is insufficient [27]. Thus, SIRT activity depends on NAD^+^ availability, making these enzymes sensors of cellular energy status that influence DNA repair, chromatin state, metabolic regulation, stress resistance, epigenetic modulation, and aging [24,25,28,29]. SIRT1 and SIRT6 are mainly nuclear and maintain genome stability, regulate DNA repair, and chromatin remodeling [25]. For instance, NAD^+^ deficiency impairs SIRT6 function, leading to telomere shortening. Notably, this condition can be rescued by NAD^+^ precursors such as NMN, supporting the role of NAD^+^ fluctuations in compromising genome integrity and accelerating aging [30]. SIRT1, SIRT3, and SIRT6 participate in the regulation of mitochondrial homeostasis and oxidative stress responses. SIRT1 and SIRT3 enhance mitochondrial function and ROS detoxification through the deacetylation of key metabolic regulators, including PGC-1α, FOXO transcription factors, and mitochondrial antioxidant enzymes. SIRT6 contributes to oxidative stress regulation via redox-related transcriptional pathways and antioxidant gene expression. In specific contexts, SIRT1-mediated deacetylation of p53 modulates stress-induced apoptosis and cell-cycle arrest, linking cellular NAD^+^ levels to redox balance and cell fate decisions [31].

PARPs catalyze mono- or poly(ADP-ribosyl)ation (PARylation), transferring ADP-ribose units from NAD^+^ to target proteins or themselves [32,33]. PARP1, the most well-characterized member of the family, detects DNA damage and catalyzes PARylation, producing NAM as a byproduct and linking DNA repair to cellular NAD^+^ metabolism [32,34]. PARP1 detects DNA damage through its zinc finger domains and, upon binding to damaged DNA, undergoes a conformational change that enables NAD^+^ binding and PARylation activity. This recruits DNA repair proteins to facilitate pathways such as Base Excision Repair (BER), Non-Homologous End Joining (NHEJ), and Homologous Recombination (HR). While moderate PARP1 activation promotes repair, excessive activation can deplete NAD^+^ and become cytotoxic [33,34,35]. Indeed, excessive PARP1 activity reduces glycolysis, impairs ATP production, and triggers parthanatos, a cell-death mechanism associated with mitochondrial AIFM1 release [36,37,38]. Restoration of NAD^+^ pools via the salvage pathway, through NAMPT and NMNAT1 activity, is essential for continuous PARP and SIRT function [39,40,41]. The nuclear isoform NMNAT1 is recruited to specific DNA promoter sites, where it synthesizes NAD^+^ locally for PARP1, ensuring a ready supply of co-substrate and regulating gene expression. This mechanism may also support PARP1 activity during DNA damage repair [41].

cADPRSs are surface glycoproteins involved in calcium signaling and NAD^+^ metabolism. Among these, CD38 is a multifunctional cell-surface protein that serves as both a receptor and an ectoenzyme. It catalyzes the hydrolysis of NAD^+^, releasing NAM, and generating the calcium-mobilizing messengers adenosine diphosphate ribose (ADPR) and its cyclic form (cADPR). CD38 is predominantly expressed on immune cells, including T cells, natural killer (NK) cells, and dendritic cells [42]. Its expression increases with age and inflammation due to cell senescence, contributing to NAD^+^ decline, metabolic stress, and inflammaging [43,44]. Altogether, these metabolic mechanisms provide the biological basis for the effects of NAD^+^ precursors in the skin.

## 3. Biological Role of NAM and NAD^+^ Precursors in the Skin

Cellular stressors, including DNA damage, oxidative stress, and chronic inflammation, trigger persistent DNA damage responses and genomic instability, driving cells toward senescence [45]. The progressive accumulation of senescent cells, together with the age-related decline in autophagy, a critical process for clearing damaged organelles and protein aggregates, contributes to tissue dysfunction, thus promoting aging and increasing cancer risk [11,12]. Throughout the lifespan, NAD^+^ levels decline in several tissues, including the nervous system, skeletal muscle and skin, with downstream activation of pro-senescent programs, chronic low-grade inflammation and impaired stress resistance.

**Figure 2:**
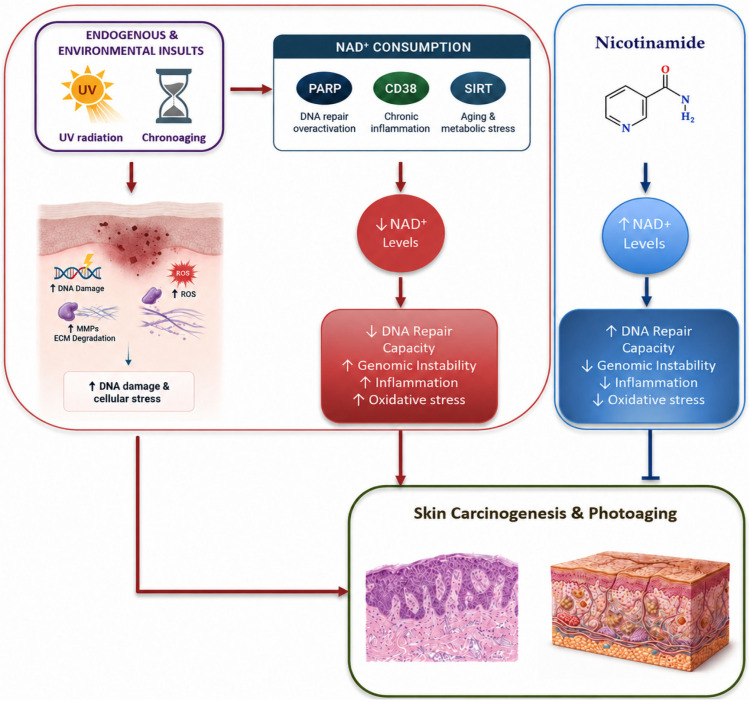
NAD^+^ depletion as a central mechanism linking photoaging and skin carcinogenesis: the protective role of nicotinamide. Ultraviolet radiation, oxidative stress, and aging promote NAD^+^ consumption through PARP, CD38, and sirtuins, leading to genomic instability, impaired DNA repair, and tissue damage. These processes contribute to both photoaging and tumor development. Nicotinamide (NAM), via the salvage pathway, restores NAD^+^ levels, enhances DNA repair, reduces inflammation and oxidative stress, and counteracts these pathogenic mechanisms.

Reduced NAD^+^ bioavailability is due to both age-associated alterations in biosynthesis and increased consumption by NAD^+^-dependent enzymes such as PARPs and CD38, which are activated in response to DNA damage, oxidative stress and inflammatory signals. In the skin, UV radiation and oxidative stress accelerate senescence and extracellular matrix degradation by depleting NAD^+^. Reduced NAD^+^ availability impairs SIRT activity, decreases DNA repair, reduces autophagic flux, increases oxidative damage, and promotes a chronic pro-inflammatory senescence-associated secretory phenotype (SASP). By sustaining intracellular NAD^+^ levels in the skin, NAD^+^ precursors can preserve genome stability, mitochondrial integrity, and redox balance, thereby limiting inflammatory signaling and preventing the onset of cellular senescence. However, the biological effects of NAM should be interpreted within the dynamic regulation of NAD^+^-dependent enzymes. While NAM replenishes NAD^+^ pools, it is also a product inhibitor of sirtuins, and its intracellular accumulation may transiently suppress SIRT activity if recycling through NAMPT is insufficient. This aspect may be particularly relevant in aged or photodamaged skin, where NAMPT expression declines, potentially influencing the efficiency of NAD^+^ restoration [6,11,12,46] (Figure 2).

In vitro, NAD^+^ delivered via optimized liposomal formulations (LF NAD^+^) reduces senescence in keratinocytes and endothelial cells, highlighting its beneficial effects on skin aging [47]. However, in practical applications, NAD^+^ precursors, such as NAM, NR, or NMN, are more commonly used to support cellular NAD^+^ metabolism and skin health.

Regarding DNA damage, NAM treatment enhances DNA repair, mitigating UV-induced ATP depletion, and thus reduces cyclobutane pyrimidine dimers (CPDs) and oxidative DNA lesions (8-oxoG) in skin models [48,49]. Preclinical in vivo studies further support the effectiveness of NAM in chemoprevention of NMSCs. Studies in mouse models indicated that NAM administration mitigates UV-induced DNA damage and immunosuppression [50]. Moreover, in UV-exposed hairless mice, oral NAM administration delays the onset of cutaneous tumors and reduces tumor burden [51].

With respect to oxidative stress, NAM improves oxidative stress responses and mitochondrial function in human primary keratinocytes, three-dimensional epidermal models, and fibroblasts from aged donors. It enhances mitochondrial autophagy (i.e., mitophagy), OXPHOS complex activity, and bioenergetic efficiency, and therefore attenuates ROS accumulation and counteracts cellular senescence [52,53,54,55]. Furthermore, NAM supplementation may also enhance antioxidant capacity in skin cells by increasing intracellular NAD^+^ availability and thus supporting SIRT1, SIRT3 and SIRT6 activity. Through NAD^+^-dependent pathways, SIRTs can promote the expression of antioxidant enzymes such as MnSOD and catalase, partly via AMPK, Nrf2, and FOXO modulation [31,56]. In skin fibroblasts, increased SIRT6 expression strengthens antioxidant defenses via the Nrf2/HO-1 axis and suppresses pro-inflammatory NF-κB signaling, thereby attenuating photoaging phenotypes [57]. NAD^+^-dependent deacetylation by SIRT1 activates core autophagy components and promotes autophagic flux in mammalian cells [58]. In addition, an elevated NAD^+^/NADH ratio can regulate mitochondrial number and function through SIRT1-mediated mitophagy [59]. In autophagy-deficient mouse fibroblasts, hyperactivation of NAD-consuming enzymes, such as PARPs and SIRTs, leads to NAD^+^ depletion, mitochondrial membrane depolarization, and cell death. Supplementation with NAM or NR restores NAD^+^ levels and cell viability [60]. Mesenchymal stem cell-derived apoptotic bodies restore NAD^+^ and mitochondrial homeostasis via NAMPT transfer, FOXO1 deacetylation, and PINK1/PARKIN-dependent mitophagy activation in keloid xenograft and scleroderma murine models [61]. At the level of NAD^+^ metabolism, NAMPT activity is essential for maintaining intracellular NAD^+^ levels and supporting SIRT1-mediated deacetylation of p53 in response to oxidative stress induced by UV irradiation [40]. In addition, PARP1 inhibition prevents UV-induced NAD^+^ depletion, highlighting the importance of maintaining NAD^+^ availability to protect against photoaging [48]. Administration of NMN protects human skin fibroblasts from oxidative stress and photoaging by increasing NAD^+^ levels and supporting SIRT1, SIRT5, and SIRT6 activity, enhancing antioxidant defenses and slowing cellular aging [62]. In a mouse model of skin aging, NMN treatment boosts NAD^+^ levels, stimulates the NAD^+^/SIRT3 axis, promotes mitophagy, and improves mitochondrial function, delaying aging phenotypes [63]. NMN supplementation also ameliorates mitochondrial function and rescues cellular senescence in mesenchymal stem cells through the activation of the NAD^+^/SIRT3 pathway [64].

In terms of chronic inflammation, NAD^+^ status closely regulates this process through SIRT1-mediated inhibition of NF-κB, a central pro-inflammatory transcription factor. NAM supplementation reduces inflammatory signaling by indirectly inhibiting NF-κB activity. In vitro, NAM decreased the expression of pro-inflammatory cytokines such as IL-6, MCP-1, and TNF-α in UV-irradiated HaCaT keratinocytes [65]. In vivo, NR administration reduced circulating cytokines, including IL-6, IL-5, and IL-2 in aged men, promoting anti-inflammatory profiles [66]. Inflammatory cytokines secreted by senescent cells induce CD38 expression [43]. Pro-inflammatory M1-like macrophages further enhance CD38 levels and NADase activity [44]. In skin cells, CD38 inhibition combined with NAD^+^ supplementation protects against photoaging and intrinsic aging by improving mitochondrial function and SIRT activity [67].

Additional features of skin aging include extracellular matrix weakening and epidermal barrier impairment. In both non-irradiated and UVA-exposed dermal fibroblasts, NAM and its derivatives increased the expression of structural proteins such as elastin and fibrillin-1/2. They also directly inhibited matrix-degrading enzymes, including MMP 1, MMP 3, MMP 9, and elastase, supporting maintenance of the extracellular matrix [68]. In cultured human keratinocytes, NAM upregulates the synthesis of key barrier lipids, including ceramides, sphingomyelin and glucosylceramide fractions, free fatty acids, and cholesterol, and increases differentiation-associated proteins such as involucrin and filaggrin, supporting the formation of competent corneocytes for improving epidermal permeability barrier [69].

Overall, these mechanisms may underlie the anti-aging and chemopreventive effects of NAM and other NAD^+^ precursors in the skin. However, it should be noted that many preclinical studies employ NAM concentrations that may exceed those achievable in human skin following systemic administration. In addition, commonly used models, such as immortalized keratinocyte cell lines or acute UV exposure in animal models, may not fully replicate the complexity of chronic human photodamage. These limitations should be considered when translating mechanistic findings into clinical expectations.

## 4. Clinical Evidence: NAM and NAD^+^ Precursors in Aging

Aging is consistently associated with a progressive decline in NAD^+^ availability across multiple tissues and downstream activation of pro-senescent programs, chronic low-grade inflammation and impaired stress resistance [70,71,72]. Preclinical studies showed that the restoration of NAD^+^ levels through vitamin B3 derivatives can counteract key hallmarks of aging, including mitochondrial dysfunction, inflammaging, and defective DNA repair, thereby improving tissue homeostasis and function [73]. These findings provided a mechanistic rationale for exploring NAD^+^-restoring strategies in anti-aging clinical settings [74].

### 4.1. Systemic NAM and NAD^+^ Precursors and Anti-Aging Effects

The clinical evidence in humans, although still limited, supports the ability of oral NAD^+^ precursors (i.e., NR, NMN and NAM) to increase systemic NAD^+^ availability and modulate selected biological and functional outcomes in middle-aged and older adults. Most available studies remain early-phase and are primarily focused on metabolic and physiological readouts rather than validated dermatologic endpoints.

The available clinical evidence on systemic NAD^+^ precursors is summarized in Table 1, which provides an integrated overview of study design, endpoints, statistical significance, and key limitations, allowing a structured interpretation of the heterogeneous findings.

NR has been shown to increase whole-blood and PBMC NAD^+^ levels in a dose-dependent manner, with reported increases of approximately 1.3–2.7-fold after short-term supplementation up to 1000 mg/day, along with changes in downstream NAD^+^ metabolites [75,76]. These findings confirm its oral bioavailability and capacity to enhance systemic NAD^+^ pools in humans.

NMN has been more extensively evaluated in randomized and early-phase clinical studies. Across different populations, supplementation (250–300 mg/day for 10–12 weeks) consistently increases circulating NAD^+^ levels without major safety concerns [77,78,79]. Higher-dose and short-term studies further support good tolerability and dose-dependent metabolic effects [80,81,82]. Beyond biochemical changes, several trials report improvements in physical performance, metabolic parameters, and selected quality-of-life measures in middle-aged adults, although results remain heterogeneous and not primarily designed to assess aging phenotypes [78,83,84,85]. Exploratory data have also suggested potential effects on telomere length increase in PBMCs, suggesting an anti-aging action; however, these findings remain preliminary [86].

NAM is characterized by an established safety profile and long-standing clinical use, including at high oral doses (up to 3 g/day), although most data derive from non-dermatological indications [74]. Acute supplementation studies indicate that even single doses can transiently increase circulating NAD^+^ levels and modulate systemic metabolic profiles [87].

**Table 1:** Human clinical studies on NAD^+^ precursors: systemic effects on NAD^+^ biology, aging-related functional outcomes and safety.

Compound	Study/Design	Population	Intervention	Main Findings	Statistical Significance	Main Limitations	Ref.
NR	Double-blind RCT, crossover pharmacokinetic study	12 healthy adults (30–55 y)	NR up to 1000 mg/day for several weeks	Increased whole-blood and PBMC NAD^+^ (≈1.3–2.7 fold)	Significant dose-dependent metabolite changes	Small sample, short duration	[75]
NMN	Double-blind RCT, placebo-controlled metabolic study (jRCTs041200034)	30 healthy adults (20–65 y)	250 mg/day for 12 weeks	Increased NAD^+^ pathway markers (blood NAD^+^, NAMN); no clinically meaningful safety concerns.	Significant increase in NAD^+^ markers (*p* < 0.001)	No clinical aging endpoints	[77]
NMN	Double-blind RCT, placebo-controlled study (UMIN000036321)	42 older men (≥65 y)	250 mg/day for 12 weeks	Increased circulating NAD^+^ metabolites; acceptable tolerability; improved gait speed, grip strength	Significant increase in NAD^+^ metabolites (*p* < 0.05 to *p* < 0.001), improvement in gait speed (*p* = 0.033) and left grip strength (*p* = 0.019)	Male-only cohort; short follow-up; no clinical aging or long-term safety endpoints	[78]
NMN	Double-blind RCT, placebo-controlled crossover trial (NCT03151239)	25 postmenopausal prediabetic women (55–75 y)	250 mg/day for 10 weeks	Increased NAD^+^ and metabolites, improved skeletal muscle insulin sensitivity associated with enhanced muscle gene expression related to metabolism	Significant improvement in muscle insulin sensitivity (*p* < 0.05)	Highly specific population, short duration, no skin endpoints	[86]
NMN	Phase I safety study (UMIN000021309)	10 healthy men (40–60 y)	Single doses 100–500 mg; assessment over 5 h	Well tolerated; increased NAD-related metabolites (2-Py, 4 Py)	Significant dose-dependent metabolite changes (*p* < 0.05, *p* < 0.01)	Very short-term exposure	[80]
NMN	Double-blind RCT, placebo-controlled study	32 overweight/obese adults (55–80 y)	1000–2000 mg/day for 14 days	Increased NAD^+^ metabolites; safe	Significant metabolite increases with dose-related effect; no significant safety signal	Very short intervention	[81]
NMN	Double-blind RCT, placebo-controlled study (UMIN000043084)	41 healthy adults (20–65 y)	1250 mg/day for 4 weeks	NMN was well tolerated with no clinically relevant adverse events; no significant abnormalities in hematological or biochemical parameters	No significant safety-related changes reported (*p* > 0.05 across safety endpoints)	Designed for safety only; short duration; no efficacy or aging-related endpoints	[82]
NMN	Double-blind RCT, placebo-controlled study (NCT04228640)	66 healthy adults (40–65 y)	300 mg/day for 60 days	Improved physical performance (e.g., walking distance, muscle endurance) and quality-of-life related measures; increases in NAD^+^ biomarkers	Significant improvements in selected physical performance and metabolic endpoints (*p* < 0.05 for primary/selected outcomes; some secondary endpoints not significant)	Short duration, no long-term aging outcomes	[84]
NMN	Double-blind RCT, placebo-controlled study (UMIN000038097)	48 overweight/obese older adults (≥65 y)	250 mg/day for 12 weeks	Improved sleep quality, reduced fatigue, and modest improvements in physical performance measures in selected subgroups	Significant improvements in selected sleep/fatigue and physical performance endpoints (*p* < 0.05 for several outcomes; not all endpoints significant)	Heterogeneous outcomes, reliance on self-reported measures, no long-term aging endpoints	[83]
NMN	Double-blind RCT, placebo-controlled study (ChiCTR2000035138)	48 recreational runners (27–50 y)	300–1200 mg/day for 6 weeks	Improved aerobic capacity and ventilatory threshold with dose trend; no major adverse symptoms/ECG issues.	Significant improvements vs.placebo in selected ventilatory-threshold parameters (*p* < 0.05); no significant change in VO_2_max or body composition	Small sample, short duration, heterogeneous dose response, no aging or metabolic endpoints	[85]
NMN	Open-label pilot (NCT04228640)	8 healthy men (45–60 y)	300 mg/day for 30–90 days	Increased telomere length in PBMCs	Significant increase in PBMC telomere length (*p* < 0.05/0.01)	Exploratory finding due to open-label design and very small sample size.	[86]
NAM	Acute supplementation study	5 healthy adults	Single oral NAM dose (100 or 500 mg)	500 mg transiently increased blood NAD^+^ levels and altered circulating lipidomic profile; well tolerated	Significant increase in blood NAD^+^ at 12 h with 500 mg (*p* < 0.05); significant lipidomic changes reported	Very small sample size, acute exposure only, no clinically relevant endpoints	[87]

Abbreviations: NAD^+^, nicotinamide adenine dinucleotide; NAM, nicotinamide; NMN, nicotinamide mononucleotide; NR, nicotinamide riboside; PBMC, peripheral blood mononuclear cell; RCT, randomized controlled trial; VO_2_max, maximal oxygen uptake.

Overall, systemic NAD^+^ precursors consistently demonstrate the ability to restore NAD^+^ bioavailability and influence systemic aging-related pathways. However, evidence directly linking these effects to clinically significant improvements in skin aging remains insufficient, as most studies have not included validated dermatologic endpoints.

### 4.2. Topical NAM and Skin Anti-Aging Effects

Topical NAM is a well-established cosmeceutical agent with multiple biological effects relevant to skin aging and photodamage. At clinically used concentrations (approximately 4–5%), it is generally well tolerated and consistently associated with improvements in both functional and visible skin aging parameters [6,88].

A detailed synthesis of the clinical evidence on topical NAM and related derivatives is presented in Table 2, which reports study design, outcomes, statistical significance, and methodological limitations in a structured format to facilitate critical interpretation.

Randomized controlled trials in photoaged skin have shown that topical NAM improves fine lines, wrinkles, pigmentation irregularities, skin texture, and sallowness. In a pivotal split-face study, 5% NAM significantly improved multiple clinical signs of facial aging compared with vehicle after 12 weeks [89,90]. Similar benefits have been reported with 4% formulations, alone or in combination with other actives, although with variable magnitude across studies [91,92].

In addition to clinical effects, NAM improves epidermal barrier function, increasing hydration and reducing transepidermal water loss (TEWL), supporting a mechanistic link between barrier restoration and visible anti-aging outcomes [93,94,95,96]. Some studies also suggest modulation of SASP pathways in treated skin [97,98].

Several studies evaluating multi-ingredient formulations containing NAM report improvements in wrinkles, pigmentation, and overall skin quality [98,99,100,101]. However, interpretation is limited by combination products and heterogeneous study designs, which prevent attribution of effects specifically to NAM. A lipophilic derivative, myristyl nicotinate, has also demonstrated enhanced dermal delivery, increased cutaneous NAD^+^ levels, improved epidermal structure, and reduced TEWL, without evidence of photosensitization [96].

Furthermore, NAM contributes to pigmentation regulation, mainly through inhibition of melanosome transfer. Clinical evidence suggests that topical NAM improves dyschromia, a major component of photoaging, primarily by inhibiting melanosome transfer from melanocytes to keratinocytes rather than directly suppressing melanogenesis. Clinical studies show improvements in hyperpigmentation and overall skin tone, including in melasma, where efficacy appears comparable to hydroquinone but with better tolerability [102,103,104].

Emerging evidence suggests that NAM may contribute to cutaneous homeostasis through modulation of epidermal barrier function, anti-inflammatory signaling, and antimicrobial activity mechanisms that could indirectly influence the skin microenvironment and microbiome [105]. However, clinical data supporting a relevant impact of NAM treatment on the skin microbiome remain limited.

**Table 2:** Clinical evidence of topical nicotinamide (NAM) and derivatives in skin aging, pigmentation and barrier function.

Study Design	Population	Treatment	Main Outcomes	Statistical Significance	Main Limitations	Ref.
Double-blind, vehicle-controlled, split-face (left–right randomized) trial	50 women	5% NAM moisturizer vs.vehicle twice daily for 12 weeks	Reduced wrinkles, hyperpigmented spots, red blotchiness, skin yellowing, texture alterations and skin elasticity	Significant skin appearance improvements vs.vehicle for fine lines/wrinkles, hyperpigmented spots, red blotchiness, and skin sallowness. Elasticity, measured via cutometry, was improved.	Predominantly investigator-assessed clinical outcomes	[89,90]
Randomized double-blind placebo-controlled split-face trial	52 women	0.03% kinetin + 4% niacinamide (group 1) vs.niacinamide 4%(group 2) twice daily for 12 weeks	Combination treatment produced greater skin improvements than niacinamide alone	Group 1: persistent and significant reductions in spot, pore, wrinkle, and evenness counts were found at weeks 8 and 12 in group 1. A significant increase in corneal hydration status was also evident at week 12, whereas persistent decreases in erythema index were apparent at 8 and 12 weeks. Group 2: more modest improvements with significant reductions in pore and evenness counts at week 8 and wrinkle counts at week 12 were noted.	Short-term study with predominantly investigator-assessed clinical outcomes and limited generalizability.	[91]
Randomized, vehicle-controlled, split-face trial	30 women	4% niacinamide lotion vs.vehicle lotion once daily for 8 weeks	Reduced wrinkles and skin roughness	Wrinkle grade significantly decreased vs.baseline and vs.vehicle (*p* < 0.001 for key comparisons). Surface roughness improved vs.baseline (*p* < 0.01) and vs.control (*p* < 0.05).	Short follow-up duration	[92]
Multi-arm translational package: (i) clinical facial study + (ii) treated-vs-untreated arm	44 women (face); 30 women (arm biopsies)	Topical formula centered on 6% niacinamide plus hyaluronic acid fractions once daily for 8 weeks	Significant improvements in wrinkles/fine lines and skin quality scores; radiance +44% at 2 months, smoothness score +39% on average; fine lines −15% on average; transcriptomics consistent with “senomorphic” signaling (down-modulation of multiple SASP genes, such as MMP12/CXCL9 and S100A8/9).	Highly significant improvements in radiance, smoothness, homogeneity, and plumpness at 1 and 2 months (*p* < 0.0001 for most endpoints). Radiance increased significantly at 1 and 2 months (*p* < 0.0001). Skin smoothness improvements were significant at both time points (*p* < 0.0001). Reductions in fine lines were significant (*p* < 0.004), as were reductions in wrinkles (*p* < 0.004). Twenty-four aging-/SASP-related genes were significantly modulated (*p* < 0.05), with predominant downregulation (20 downregulated vs. 4 upregulated).	Effects confounded by multi-ingredient formulation	[97]
Randomized, parallel-group facial appearance study	99 (test)/97 (control) subjects	Multi-product regimen: SPF30 day lotion (5% niacinamide + peptides) + night cream (niacinamide + peptides, NPP) + wrinkle treatment (niacinamide + peptides + 0.3% retinyl propionate) vs.comparator regimen incl. 0.02% tretinoin (emollient base) + SPF30. Wrinkle treatment twice daily; day lotion + night cream daily; 8 weeks (subset continued +16 weeks)	Improved facial wrinkle appearance and photodamage parameters; NPP regimen showed greater responder rates and superior self-assessed improvements vs.tretinoin. TEWL remained stable in NPP but increased with tretinoin; erythema and dryness were higher with tretinoin.	Significant improvement in wrinkle appearance vs.baseline (*p* ≤ 0.05) in both groups; NPP superior to tretinoin for wrinkle improvement (*p* < 0.01). Higher responder rates with NPP (*p* = 0.03–0.02). Significant between-group differences favoring NPP in self-assessment outcomes at 4 and 8 weeks (*p* < 0.05). Significant between-group difference in TEWL change (*p* < 0.01).	Effects confounded by multi-active formulation	[98]
Randomized, double-blind, vehicle-controlled, split-face trial	40 women	2% gold silk sericin + 5% niacinamide + 0.1% Signaline™ vs.simple O/W emulsion twice daily for 12 weeks	Improved hydration, elasticity, and barrier-related biophysical parameters	Significant improvements versus vehicle in hydration, elasticity, barrier function, and surface topography (*p*< 0.05 for all measured biophysical parameters).	Multi-component formulation	[99]
Open-label pilot study	25 women	Night formulation: 0.5% retinol + 4.4% niacinamide + 1% resveratrol + 1.1% hexylresorcinol for 10 weeks	Improved pigmentation and wrinkle appearance from week 4 to week 10	Statistical interpretation is limited by the open-label, uncontrolled design	Uncontrolled study design	[100]
Double-blind, randomized, split-face, vehicle-controlled (post-procedure)	24 patients	2% adipocyte-derived MSC-conditioned medium + 2% niacinamide vs.vehicle for 3 weeks after fractional ablative CO_2_ laser therapy	Reduced wrinkles and pigmentation after laser treatment	Reduced wrinkle index (*p* = 0.036) and melanin index (*p* = 0.043) vs.control	Small sample size and short follow-up; multi-component formulation	[101]
Randomized, double-blind, placebo-controlled clinical program (photodamaged skin; multi-study report)	96 subjects	Myristyl nicotinate (MN) topical (1–5% across studies) vs.placebo formulations daily or twice-daily applications for ~12 weeks	Increased epidermal NAD levels (+25%), stratum corneum thickness (~+70%) and epidermal thickness (~+20%) vs.placebo; reduced TEWL (~20%) on cheeks and arms	Significant increases in skin NAD (*p* = 0.001), stratum corneum thickness (*p* = 0.0001), epidermal thickness (*p* = 0.001), epidermal renewal (*p* ≤ 0.003), and significant TEWL reductions on cheeks (*p* = 0.012) and arms (*p* = 0.017)	Evaluated NAM derivative	[96]
Controlled clinical studies	18 women with hyperpigmentation; 120 women with facial tanning	Topical 2–5% NAM formulations for 4 weeks	Reduced hyperpigmentation and improved skin lightness in treated groups; inhibition of melanosome transfer was demonstrated mechanistically	Significant improvements in pigmentation parameters versus baseline and/or control conditions after 4 weeks (*p* < 0.05 where reported)	Heterogeneous study populations and endpoints; short treatment duration; limited mechanistic–clinical correlation;	[102]
Double-blind randomized clinical trial	27 patients with melasma	4% NAM vs.4% hydroquinone (HQ) for 8 weeks	Comparable improvement in melasma severity, reduction in inflammatory infiltrate, and solar elastosis, with better tolerability for NAM	No significant difference NAM vs.HQ; fewer adverse effects with NAM (18% with NAM vs.29% with HQ).	Small sample size and short treatment duration	[104]

Abbreviations: CO_2_, carbon dioxide; HQ, hydroquinone; MSC, mesenchymal stem cell; NAM, nicotinamide; NPP, niacinamide/peptides/retinyl propionate regimen; SASP, Senescence-Associated Secretory Phenotype; TEWL, transepidermal water loss.

Therefore, topical NAM is supported by consistent clinical evidence for improvement in multiple features of skin aging, although study heterogeneity, short follow-up periods, and frequent use of combination formulations are important limitations.

## 5. Clinical Evidence: NAM in NMSC Prevention

Since DNA photodamage is central to NMSC development, NAM-mediated protection of skin cells against UV-induced ATP depletion, DNA damage, and pro-inflammatory responses underlies its potential role in counteracting tumor development and provides a rationale for its use as a chemopreventive agent in clinical studies [3].

### 5.1. Systemic NAM and Chemoprevention

A structured overview of these studies is provided in Table 3, which summarizes efficacy endpoints, statistical significance, and methodological limitations to allow direct comparison across heterogeneous clinical settings.

Clinical evidence for systemic NAM in NMSC prevention is primarily derived from high-risk populations, especially patients with a history of multiple keratinocyte carcinomas. The pivotal phase III ONTRAC trial demonstrated that oral NAM (500 mg twice daily) reduced the incidence of new NMSCs and AKs over 12 months, with a more pronounced effect on SCC compared with BCC [106]. This trial, conducted in 386 immunocompetent patients, also suggested that the chemopreventive effect is time-dependent, as benefits were lost after discontinuation, indicating the need for continuous administration to maintain protection. Dose selection was supported by a preceding phase II randomized trial showing a greater reduction in AKs with twice-daily dosing compared with once-daily administration [107]. Additional mechanistic data suggest that NAM supplementation may modulate SIRT1 activity in PBMC through enhanced nuclear translocation rather than changes in protein expression, providing a potential link between metabolic modulation and DNA repair capacity [108].

Real-world evidence from large retrospective cohorts further supports a reduction in subsequent skin cancer risk, particularly when NAM is initiated early after the first diagnosis [109]. However, findings across systematic reviews and meta-analyses remain inconsistent, with some reporting significant reductions in NMSC and SCC in high-risk populations or organ transplant recipients, while others show no statistically significant benefit when heterogeneous datasets are combined [110,111]. Similarly, data in solid organ transplant recipients remain inconclusive, with a randomized trial (ONTRANS) failing to demonstrate significant benefit, likely due to early termination and limited statistical power [112].

Recent metabolomic studies have raised additional considerations regarding long-term systemic effects of vitamin B3 supplementation, particularly in relation to vascular inflammation and endothelial activation associated with niacin metabolites (e.g., 4-Py derivatives) [113]. However, these findings derive largely from high-dose niacin exposure in cardiovascular populations, and their relevance to dermatologic dosing of NAM remains uncertain. Indeed, the metabolomic signal was observed in cohorts receiving pharmacological niacin doses (≈1.5–2 g/day), substantially higher than those used for skin cancer chemoprevention (500–1000 mg/day), and did not distinguish between nicotinic acid and NAM, which have distinct metabolic pathways and physiological effects. Furthermore, available retrospective data have not demonstrated a significant association between nicotinamide use at dermatologic doses and cardiovascular events [109].

**Table 3:** Summary of clinical evidence of nicotinamide (NAM) in skin cancer protection.

Study Type	Population	Treatment	Main Outcomes	Statistical Significance	Main Limitations	Reference
Phase III randomized, placebo-controlled trial (ONTRAC)	386 high-risk patients with previous NMSC	Oral NAM 500 mg twice daily for 12 months	Reduced incidence of new non-melanoma skin cancers, mainly SCC and AK	23% reduction in NMSC (95% CI: 4–38%, *p* = 0.02); stronger effect for SCC (95% CI: 0–51%, *p* = 0.05); AK reduction (0.001 < *p* < 0.01)	No long-term post-intervention follow-up after discontinuation	[106]
Phase II double-blinded randomized controlled trial	Patients with AKs (n ≈ 74–114 across dosing arms, depending on protocol phase)	Oral NAM 500 mg once or twice daily	Dose-dependent reduction in AKs, favoring twice-daily dosing	Greater AK reduction with BID dosing (*p* = 0.005) than OD dosing (*p* = 0.05)	Small sample size; short duration; surrogate endpoint (AK rather than cancer incidence)	[107]
Retrospective cohort study	>33,000 patients with a prior history of NMSC	Oral NAM 500 mg twice daily	Reduced risk of subsequent non-melanoma skin cancers in a real-world setting	14% overall reduction (HR 0.86; 95% CI 0.82–0.89); 54% reduction when started after first NMSC (HR 0.47; 95% CI 0.23–0.97); reduction for SCC (HR 0.78; 95% CI 0.75–0.82) and BCC (HR 1.00; 95% CI 0.96–1.05)	Observational design; residual confounding; lack of randomization	[109]
Systematic review and meta-analysis	High-risk patients/organ transplant recipients (multiple pooled studies)	Oral NAM	Reduced incidence of BCC and SCC; increased gastrointestinal (GI) adverse events	Significant reduction in BCC (RR 0.46; 95% CI 0.22–0.95) and SCC (RR 0.48; 95% CI 0.26–0.88); ↑ GI adverse effects (RR 1.78; 95% CI 1.30–2.45)	Variable study quality; pooled populations with differing baseline risk	[110]
Systematic review and meta-analysis	Mixed populations (multiple RCTs and observational datasets)	Oral NAM	No significant reduction in SCC, BCC, or total NMSC	No significant reduction in SCC (RR 0.81; 95% CI 0.48–1.37), BCC (RR 0.88; 95% CI 0.50–1.55), or total NMSC (RR 0.82; 95% CI 0.61–1.12)	Clinical and methodological heterogeneity; differences in included study designs and populations	[111]
Phase III randomized, placebo-controlled trial (solid organ transplant recipients; ONTRANS)	158 solid organ transplant recipients	Oral nicotinamide 500 mg twice daily vs.placebo for 12 months	No significant reduction in NMSC cancers, including SCC, BCC, or AK, in immunosuppressed transplant recipients	No significant difference between NAM and placebo for SCC, BCC, or total NMSC (rate ratio ~1.0; *p* not significant)	Early trial termination; underpowered study; immunosuppressed population limiting comparability with prior RCTs	[112]

Abbreviations: AK, actinic keratosis; BCC, basal cell carcinoma; BID, twice daily; CI, confidence interval; HR, hazard ratio; NMSC, non-melanoma skin cancer; NAM, nicotinamide; OD, once daily; RCT, randomized controlled trial; RR, risk ratio; SCC, squamous cell carcinoma.

Overall, systemic clinical evidence for NAM in chemoprevention is heterogeneous and is best interpreted in the context of study design, patient risk stratification, and immune status. Larger, well-designed trials are therefore needed to clarify its efficacy across different risk groups and generalizability to broader populations.

### 5.2. Topical NAM and Local Photoprotection

In addition to systemic administration, topical NAM has been investigated as a strategy to enhance resistance to UV-induced immunosuppression under physiologically relevant conditions. Clinical evidence on topical NAM and local photoprotection is summarized in Table 4.

Early randomized double-blind studies using solar-simulated UV exposure demonstrated that topical NAM (0.2–5%) significantly reduced UV-induced suppression of delayed-type hypersensitivity responses, with protective effects observed after both single and repeated applications [114]. NAM attenuated immunosuppressive effects induced by both UVB and longwave UVA radiation, supporting its role as a complementary form of “biological photoprotection,” particularly against UVA-related damage that is less effectively blocked by conventional sunscreens. Topical NAM also increased the expression of oxidative phosphorylation-related enzymes in UV-exposed keratinocytes, consistent with improved cellular energy metabolism during UV stress [114].

Further evidence of local immunomodulatory activity derives from studies using methyl aminolevulinate photodynamic therapy (PDT) as a model of photo-induced immunosuppression. In a randomized controlled study, topical 5% NAM significantly reduced PDT-induced immunosuppression, with effects comparable to oral administration [115]. In addition, a phase II double-blind study using AKs as a surrogate endpoint reported that topical 1% NAM lotion applied twice daily for 6 months significantly reduced AK counts in sun-damaged patients compared with vehicle treatment [116].

In conclusion, available data suggest that topical NAM may enhance cutaneous resistance to photo-induced immune suppression and early UV-related damage. However, current evidence is still limited by relatively small studies, short follow-up periods, and the use of surrogate immunologic or precancerous endpoints rather than long-term skin cancer outcomes.

## 6. Discussion

Dermatologists have long recognized that chronically sun-damaged skin is characterized by both accelerated aging and an increased risk of carcinogenesis, but these processes are often approached as clinically distinct entities. The evidence reviewed here supports an integrated pathophysiological model in which photoaging and carcinogenesis converge on shared metabolic alterations, particularly involving NAD^+^ homeostasis.

### 6.1. A Shared Metabolic Mechanism: NAD^+^ as a Central Node

Chronic UV exposure drives both skin aging and carcinogenesis through overlapping molecular pathways, with progressive NAD^+^ depletion emerging as a shared upstream mechanism. NAD^+^-dependent enzymes, including PARPs, sirtuins, and CD38, regulate DNA repair, metabolic homeostasis, and inflammatory responses, and their activity declines with age and cumulative photodamage. In this context, NAM and other precursors act by supporting NAD^+^ salvage rather than targeting individual downstream processes, providing a unifying mechanistic basis linking anti-aging and chemopreventive effects. However, a strong biological rationale alone is not sufficient to support clinical recommendations.

### 6.2. Clinical Evidence: NAM Selective Topical Effects and Systemic Benefit

The anti-aging clinical evidence is strongest for topical NAM, which consistently improves photoaging parameters across multiple controlled trials [89,90,91,92,97]. Systemic NAM has demonstrated efficacy in reducing NMSC incidence in high-risk populations [106], particularly for SCC, although results remain heterogeneous across studies. This variability likely reflects differences in study populations, especially between immunocompetent and immunosuppressed individuals [110,111]. Compared with existing chemopreventive options, NAM offers a favorable balance between efficacy and tolerability, filling a niche as a safe, systemic alternative to retinoids, which are limited by significant toxicity. Although its effect size is more modest, NAM represents a practical option for long-term prevention, particularly in immunocompetent patients unable to tolerate retinoids.

In contrast, other NAD^+^ precursors, such as NMN and NR, despite robust preclinical metabolic effects, currently lack evidence based on validated dermatologic endpoints, highlighting a gap between systemic NAD^+^ modulation and clinically measurable skin outcomes. This limitation, together with their substantially higher cost, less established long-term safety profiles, and variable quality control in the supplement market, does not currently support their routine use in dermatologic practice.

### 6.3. Limitations, Safety Considerations, and Future Directions

Interpretation of the current clinical evidence requires several important considerations, largely related to methodological heterogeneity and limited long-term data. Many available studies are limited by relatively small sample sizes, short follow-up periods, heterogeneous patient populations, and variability in formulations, dosing regimens, and evaluated endpoints. In anti-aging studies, clinically meaningful dermatologic outcomes are often secondary endpoints, and many trials rely on subjective measures rather than standardized skin-aging biomarkers. In addition, several topical studies evaluated multi-ingredient formulations, making it difficult to attribute observed effects specifically to NAM itself.

Similarly, chemoprevention studies differ substantially in study design, baseline cancer risk, immunological status, and outcome assessment, which may partly explain the heterogeneity of reported results. The limited number of long-term randomized trials, particularly in immunosuppressed populations, further restricts the generalizability of current findings. Moreover, many mechanistic observations derive from preclinical models employing supra-physiological NAM concentrations or acute UV exposure systems that may not fully replicate the complexity of chronic human photodamage. In addition, the requirement for continuous administration to maintain chemopreventive effects highlights the need for sustained adherence in real-world settings.

Long-term safety considerations also warrant attention. Recent metabolomic studies [105] have raised questions regarding the potential cardiovascular effects of niacin metabolites, particularly in relation to vascular inflammation. Although these findings derive mainly from high-dose exposure and populations with pre-existing cardiovascular disease, they underscore the importance of prospectively evaluating the long-term safety of NAM at dermatologic doses.

Future research should prioritize large, stratified randomized controlled trials to better define efficacy across different patient populations, including immunocompetent and immunosuppressed individuals, and to establish the optimal timing of treatment initiation. Head-to-head comparisons between NAM and other NAD^+^ precursors, such as NR and NMN, using validated skin-specific endpoints, are also needed to guide clinical decision-making. In parallel, combined therapeutic strategies, such as the integration of systemic and topical NAM, should be formally evaluated, while targeting NAD^+^ consumption pathways (e.g., CD38 inhibition) may represent a complementary approach to enhance efficacy. Advances in delivery systems may further improve cutaneous NAD^+^ bioavailability, and the influence of the gut microbiome on NAD^+^ precursor metabolism may help explain interindividual variability in treatment response.

From a translational perspective, the interaction between NAM metabolism and NAD^+^-dependent enzymes may influence clinical response. NAM, as a product of sirtuin-catalyzed reactions, can act as a non-competitive inhibitor when locally accumulated [3]. Moreover, age-related changes in NAD^+^ turnover and recycling capacity may affect treatment efficacy, particularly in elderly individuals with chronically photodamaged skin, and should be considered when optimizing dosing strategies.

Interindividual variability in susceptibility to photoaging and skin carcinogenesis likely reflects genetic factors influencing key biological processes. Single-nucleotide polymorphisms (SNPs) have been increasingly recognized as modulators of cancer risk, as they may affect individual responses to environmental stressors, immune surveillance, and genomic stability, particularly under chronic UV exposure [117]. Consistently, genome-wide association studies have identified multiple SNPs associated with NMSC risk in genes involved in pigmentation (e.g., MC1R, ASIP, SLC45A2), immune regulation (e.g., IFIH1, CCR5), and tumor suppression pathways (e.g., TP53, CDKN2A), while polymorphisms in DNA repair genes such as XPC, ERCC2, and FANCA have been linked to reduced repair efficiency and increased UV-related skin cancer risk [118,119]. Our recent findings also support the relevance of genetic variability in genes involved in NAD^+^ metabolism, such as NNMT, in NMSC susceptibility [120], reinforcing the potential contribution of host genetic background to interindividual differences in disease risk and prevention response. Beyond inherited variability, dynamic age-related changes further contribute to interindividual differences in NAD^+^ biology. Age-related alterations in NAD^+^ homeostasis, including variability in enzymes involved in NAD^+^ salvage and consumption such as NAMPT, CD38, and PARPs, may further influence cellular resilience to chronic UV-induced damage and the biological response to NAD^+^-restoring strategies [121]. Although direct evidence linking genetic variants to differential response to NAM is currently lacking, these observations support the rationale for future personalized preventive approaches integrating genetic and metabolic profiling.

## 7. Conclusions

Overall, NAM represents a mechanistically supported and clinically relevant intervention targeting NAD^+^ depletion as a common pathogenic mechanism underlying both skin aging and carcinogenesis. While current evidence supports its use in selected settings, further high-quality, skin-focused studies are required to more definitively establish its role in dermatological clinical practice. In the meantime, NAM may be considered an adjunctive option for high-risk patients, particularly for secondary prevention.

## Figures and Tables

**Table 4:** Clinical and experimental evidence of topical nicotinamide (NAM) in UV-induced immunosuppression and early photodamage.

Study Type	Population	Treatment	Main Outcomes	Statistical Significance	Main Limitations	Reference
Randomized, double-blind, vehicle-controlled UV irradiation studies	Healthy Mantoux-positive volunteers (four studies)	Topical NAM 0.2–5% before and/or after solar-simulated UV exposure	Reduced UV-induced suppression of delayed-type hypersensitivity responses by ~50%; attenuation of both UVB- and UVA-induced immunosuppression; increased expression of oxidative phosphorylation-related enzymes in UV-exposed keratinocytes	Significant reduction in UV-induced immunosuppression compared with vehicle (approximately 50%; *p* < 0.05 across experimental conditions)	Experimental short-term UV model; surrogate immunologic endpoints rather than clinical cancer outcomes	[114]
Randomized controlled study using methyl aminolevulinate photodynamic therapy (PDT) model	20 healthy Mantoux-positive volunteers	Topical 5% NAM vs.vehicle after PDT	Reduced PDT-induced immunosuppression by ~59%; effects comparable to oral NAM	Significant reduction in immunosuppression versus control (*p* < 0.0001)	Short-term experimental model; no long-term clinical endpoints	[115]
Randomized, double-blind, placebo-controlled trial	30 sun-damaged patients with actinic keratoses (AKs)	Topical 1% NAM lotion twice daily for 6 months	Reduced AK counts on face, scalp, and forearms	AK reduction: 22% with NAM vs.10% with vehicle (*p* = 0.04)	Small sample size; surrogate endpoint (AK rather than NMSC incidence)	[116]

Abbreviations: AK, actinic keratosis; NAM, nicotinamide; PDT, photodynamic therapy; UVA, ultraviolet A; UVB, ultraviolet B.

## Data Availability

No new data were created or analyzed in this study. Data sharing is not applicable to this article.
